# Association between Short Stature at Grade 1 and Permanent Teeth Caries at Grade 6 in Elementary School Children in Japan: A Population-Based Cohort Study

**DOI:** 10.3390/ijerph21010105

**Published:** 2024-01-17

**Authors:** Ayako Suzuki, Yukako Tani, Tatsuhiko Anzai, Aya Isumi, Satomi Doi, Takuya Ogawa, Keiji Moriyama, Takeo Fujiwara

**Affiliations:** 1 Department of Maxillofacial Orthognathics, Graduate School of Medical and Dental Sciences, Tokyo Medical and Dental University, Tokyo 113-8519, Japan; a-suzuki.mort@tmd.ac.jp (A.S.); t-ogawa.mort@tmd.ac.jp (T.O.); k-moriyama.mort@tmd.ac.jp (K.M.); 2 Department of Global Health Promotion, Graduate School of Medical and Dental Sciences, Tokyo Medical and Dental University, Tokyo 113-8519, Japan; tani.hlth@tmd.ac.jp; 3 Department of Biostatistics, M&D Data Science Center, Tokyo Medical and Dental University, Tokyo 113-8519, Japan; tanzai.dsc@tmd.ac.jp; 4 Department of Health Policy, Graduate School of Medical and Dental Sciences, Tokyo Medical and Dental University, Tokyo 113-8519, Japan; isumi.hlth@tmd.ac.jp (A.I.); doi.hlth@tmd.ac.jp (S.D.); 5 Department of International Health, Bloomberg School of Public Health, Johns Hopkins University, Baltimore, MD 21218, USA

**Keywords:** dental caries, short stature, children

## Abstract

Short stature in children is a marker of low nutritional status and has been suggested to be associated with dental caries. However, longitudinal studies on this topic are scarce. Data from a longitudinal study of elementary school children in Adachi City, Tokyo, Japan, were analyzed. In 2015, caregivers of children at grade 1 answered questionnaires, and information on dental caries and height measured at school health checkups was merged and followed to grade 6 (N = 3576; follow up rate = 83.3%). The association between short stature at grade 1 (−2.01 standard deviation (SD)–−3.00 SD, or <−3.00 SD in height-for-age according to the World Health Organization criteria) and the number of decayed, missing, or filled permanent teeth (DMFT) at grade 6 was examined using multivariable Poisson regression with robust standard error. After adjusting for confounders, children with a short stature at grade 1 had a higher DMFT number at grade 6: the mean ratios (95% confidence interval) were 1.17 (0.89–1.54) and 2.18 (1.03–4.64) for children with a height-for-age −2.01 SD–−3.00 SD, and those with a height-for-age < −3.00, respectively. Short stature at grade 1 could be a marker of future dental caries in the permanent teeth at grade 6.

## 1. Introduction

Dental caries is one of the most common chronic diseases worldwide [1]. Approximately half of children worldwide have dental caries in their permanent teeth [2]. In Japan, national school surveillance data from 2021 show that approximately 40% of Japanese elementary school children have experienced dental caries [3]. Dental caries contributes to limitations in the oral function and emotional well-being of children [4]. As restorative dental caries treatments sometimes require the retreatment of secondary dental caries [5], it is crucial to detect and address risk factors, including social, behavioral, and biological factors, in children [6].

Short stature in childhood is a marker of long-term undernutrition [7]. A previous review indicated an association between malnutrition and dental caries in primary teeth [8]. However, longitudinal studies on permanent teeth are limited [8]. Undernutrition during tooth germ formation and calcification are risk factors for dental caries [9]. The calcification of deciduous teeth begins during the fetal period, whereas the calcification of permanent teeth begins after birth [10]. Thus, undernutrition after birth, captured by stature, can be associated with dental caries in the permanent teeth. More specifically, short stature at grade 1 in elementary school, which can be a marker of undernutrition during permanent tooth calcification except for the second and third molars, can be a risk factor for dental caries at grade 6 in elementary school children in Japan.

To our knowledge, three longitudinal studies have investigated the association between short stature in childhood and dental caries in permanent teeth [9,11,12]. A study following 94 children aged 6–11 months in Peru in 1986 for six years reported that children with a single, moderate malnutrition episode, measured by height and weight during the first year of life, were at a higher risk of increasing dental caries in permanent teeth by approximately 1.5 times, although no confounders were adjusted [9]. Another study was carried out in a population-based birth cohort study that started in 1993 in Brazil [11]. This study followed 339 children aged 1, 4, 6, and 12 years. The height-for-age deficit at the age of one was related to a permanent caries prevalence at the age of 12 by 1.5 times after adjusting for confounders, including the mother’s educational attainment and children’s sex [11]. Finally, 83 of 121 children aged 7–9 years in Peru in 2002 were re-examined after 3.5 years of follow-up. The study concluded that short stature was associated with a higher risk of caries increment in permanent teeth by 1.61 times after adjusting for age, sex, sugary snacks between meals, oral hygiene, and baseline caries experience [12]. While these studies suggest an association between short stature and dental caries in permanent teeth, no study has been conducted in developed countries, such as Japan. In developed countries, many people lack micronutrients even though they have sufficient food [13]. Undernutrition in Japan is different from that in developing countries: in Japan, some children in low-income, dual-income, or single-parent families tend to eat prepared meals such as food in convenience stores or fast food, eat out more often, or skip meals, resulting in a low-vegetable, low-nutrient, and unbalanced diet [14]. Moreover, this study differs from the existing literature in that Japanese children have easy access to dental care and fluoridated toothpaste is widely available. Therefore, this study provides new insights into whether short stature is associated with permanent dental caries, even in settings with high socioeconomic status and good oral health services.

This study aimed to investigate whether a short stature at grade 1 (age 6–7 years) in elementary school is a risk factor for dental caries at grade 6 (age 11–12 years) in elementary school children in Japan.

## 2. Materials and Methods

*Study participants*

**Figure 1:**
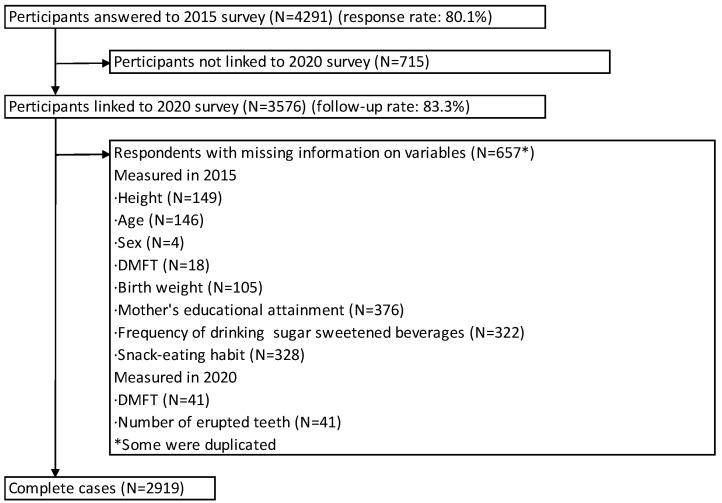
Flow chart of study participants.

Data from the Adachi Child Health Impact of Living Difficulty (A-CHILD) Study, a longitudinal study of all 69 public elementary school children in Adachi City, Tokyo, Japan, were analyzed [15]. Figure 1 shows a flowchart of the study participants. In 2015, a questionnaire was distributed to the caregivers of all first-graders in the city (N = 5355). Of these, 4291 (response rate: 80.1%) answered the questionnaire and submitted to the school using anonymous envelopes. In the same year, children’s height and dental caries were measured as part of school health checkups and linked with the survey data. The follow-up survey was conducted at grade 6 (N = 3576, follow-up rate: 83.3%) in 2020. The number of complete cases was 2919, after excluding 657 participants with missing information on variables used in the analysis, including the height of children at grade 1 (N = 149), age (N = 146), sex (N = 4), dental caries in permanent teeth at grade 1 (N = 18), dental caries in permanent teeth at grade 6 (N = 41), birth weight (N = 105), the mother’s educational attainment (N = 376), the frequency of drinking sugar-sweetened beverages at grade 1 (N = 322), snack-eating habits at grade 1 (N = 328), and the number of erupted permanent teeth at grade 6 (N = 41). All caregivers (parents or legal guardians) provided written informed consent to participate in this study. This study was approved by the Ethics Committee of the Tokyo Medical and Dental University (M2016-284), Tokyo, Japan, and the National Center for Child Health and Development, Tokyo, Japan.

*Dental caries in permanent teeth*

In Japan, school health checkups, including dental examinations, are conducted annually. The children were examined by school dentists under sufficient light in the classroom. The examinations were performed using a dental mirror and ball-ended probe. The dentists followed the standard procedures and guidelines to record any visible caries [16]. Each permanent tooth was evaluated for sound, decayed, treated, missing, or initial caries [16]. The number of decayed, missing, and filled permanent teeth (DMFT) was calculated at grade 1 and grade 6. The number of DMFT at grade 1 was used as a covariate. The number of DMFT at grade 6 was used as the outcome variable.

*Height of children*

We used the height of the children at grade 1, measured during school health checkups, as an exposure variable. It was measured barefoot by a school teacher using a height-measuring device [16]. Because the method of measuring height is simple and teachers measure many children’s heights each year, inter-examiner error is unlikely to occur. Each children’s height-for-age was classified into ≥−2.00 SD, −2.01 SD–−3.00 SD, and <−3.00 SD according to the WHO Child Growth Standards 2007 for boys and girls aged 5–19 years [17]. The WHO Child Growth Standards criteria depict normal growth under optimal environmental conditions and can be used to assess children regardless of ethnicity, socio-economic status and type of feeding. Since Japan has a relatively shorter height than the global average [18], −2.01 SD–−3.00 SD and <−3.00 SD are divided. Children whose height-for-age was −2.01 SD–−3.00 SD or <−3.00 SD were defined as having a short stature.

*Covariates*

We measured the basic demographics of the children (children’s sex and age in months) with respect to potential covariates. The questionnaire survey to caregivers assessed the following covariates: birth weight (<2500 g, 2500–3999 g, and ≥4000 g), annual household income at grade 1 (JPY <3.0, 3.0–5.9, 6.0–9.9, ≥10.0 million, and unknown; JPY 110 JPY ≈ USD 1), the mother’s educational attainment (less than high school, junior college or technical school, university or more, or others/unknown), the frequency of drinking sugar-sweetened beverages at grade 1 (every day or less than once a day), and snack-eating habits at grade 1 (controlled by caregivers or not controlled by caregivers). In the questionnaire of annal household income, the option “unknown” was given to keep the option to not answer on household income, and those who chose this option were placed in the “unknown” category. In the questionnaire about the mother’s educational attainment, the options of “unknown” and “other” were given, and those who chose these options were collapsed as the “other/unknown” category. The answer was categorized as a missing value if it was not filled in. The number of decayed, missing, or filled permanent teeth (DMFT) at grade 1 (i.e., baseline caries status) and the number of erupted permanent teeth at grade 6 (number of teeth at risk) were assessed by school dentists.

*Statistical analysis*

First, we compared the baseline demographic of complete cases (N = 2919) whose height-for-age values were ≥−2.00 SD, −2.01 SD–−3.00 SD, and <−3.00 SD according to the WHO Child Growth Standards 2007, using a t-test for continuous independent variables and a chi-square test for categorical independent variables.

Multivariable Poisson regression with robust standard error was used to examine the association between short stature at grade 1 and permanent dental caries (DMFT) at grade 6. Three models were constructed: a model adjusted for age, sex, the number of erupted teeth at grade 6, and the number of DMFT at grade 1 (model 1); a model further adjusted for birth weight (model 2); a model further adjusted for socioeconomic status with annual household income at grade 1 and the mother’s educational attainment, as well as dietary behaviors related to oral health with the frequency of drinking sugar-sweetened beverages at grade 1 and snack-eating habits at grade 1 (model 3). The number of erupted teeth at grade 6 and the number of DMFT at grade 1 were adjusted in all models because they were significantly different among all exposure groups. Models 2 and 3 were constructed to control potential confounders. Missing information for participants was imputed via multiple imputation with chained equations. Twenty datasets were created. As shown in Supplementary Table S1, the selection bias due to missing information was reduced through imputation. The STATA software (version 17.0; Stata Corp LP, Lakeway, TX, USA) was used for all the analyses.

## 3. Results

Among the 2919 participants without any missing information (i.e., complete cases), their height varied from 101.7 to 135.7 cm (mean (SD) = 116.4 (4.9)), and 380 (13.05%) children were short-statured at grade 1 (the number of children with height-for-age −2.01 SD–−3.00 SD was 350 (12.3%), and the number of children with height-for-age <−3.00 SD was 22 (0.75%)).

Table 1 presents the demographic characteristic of the complete cases (N = 2919). The mean of the DMFT at grade 1 was 0.045 (SD = 0.30) for all participants, 0.045 (SD = 0.30) for children with a height-for-age of ≥−2.00 SD, 0.034 (SD = 0.21) for children with a height-for-age of −2.01 SD–−3.00 SD, and 0.18 (SD = 0.59) for children with a height-for-age of <−3.00 SD. Regarding DMFT at grade 6, the mean of the DMFT at grade 6 was 0.42 (SD = 1.10) for all participants, 0.42 (SD = 1.09) for children with a height-for-age of ≥−2.00 SD, 0.41 (SD = 1.12) for children with a height-for-age of −2.01 SD–−3.00 SD, and 0.82 (SD = 2.02) for children with a height-for-age of <−3.00 SD. Children with short stature at grade 1 had fewer erupted permanent teeth at grade 6 ((mean (SD) = 22.3 (3.94) for children with a height-for-age of −2.01 SD–−3.00 SD, mean (SD) = 21.5 (4.38) for children with a height-for-age of <−3.00) than the other children (mean (SD) = 24.0 (3.37)). The percentage of low-birth-weight children was higher amongst short-statured children at grade 1 (21.2% for children with a height-for-age of −2.01 SD–−3.00 SD and 27.3% for children with a height-for-age of <−3.00) than in the others (7.5%). No significant differences were observed for age in months at grade 1, sex, DMFT at grade 1, annual household income, the mother’s educational attainment, the frequency of drinking sugar-sweetened beverages at grade 1, and snack-eating habits at grade 1 between the short-statured children and the others.

Table 2 shows the association between short stature at grade 1 and dental caries in the permanent teeth at grade 6 after multiple imputation (N = 3576). After adjusting for age, sex, the number of DMFT at grade 1, and the number of erupted teeth at grade 6, short stature was associated with dental caries, although it was not statistically significant (model 1; mean ratio (MR) and 95% confidence interval (CI): 1.18 (0.90, 1.55) and 2.07 (0.98, 4.36) for those with a height-for-age of −2.01 SD–−3.00 SD and <−3.00 SD, respectively). Estimates were similar when further adjusting for birth weight (model 2). After adjusting for all confounders, including socioeconomic status (annual household income and mother’s educational attainment) and dental caries-related behaviors (frequency of drinking sugar-sweetened beverages at grade 1 and snack-eating habits at grade 1), short stature at grade 1 was associated with dental caries at grade 6; model 3, MR (95% CI) were 1.17 (0.89, 1.54) and 2.18 (1.03, 4.64) for children with a height-for-age of −2.01 SD–−3.00 SD and those with a height-for-age of < −3.00 SD), respectively. A complete case analysis (N = 2919) showed similar results (Supplementary Table S2).

## 4. Discussion

Using a population-based longitudinal study, we found that a short stature of less than −3 SD at grade 1 was associated with grade 6 dental caries in the permanent teeth. This association persisted even after adjusting for potential confounders including birth weight, socioeconomic status, and oral health behaviors.

Previous longitudinal studies have reported an association between short stature and dental caries in the permanent teeth [9,11,12]. However, all of these studies were conducted in South America, and our study is the first to report the association in a developed country. The study findings showed that short stature is associated with dental caries in Japan, where populations have easy access to oral health services and fluoride toothpaste.

Of the three previous longitudinal studies on the association between short stature and permanent dental caries [9,11,12], the studies conducted in Peru in 1986 [9] and in Brazil in 1993 [11] measured the height of children at age 0 to 1 year old. The previous study conducted in Peru in 2002 [12] is similar to the present study in that it measured the height of children ages 7 to 9 using WHO Child Growth Standards 2007. As for the percentage of children with a height-for-age of < −2.00 SD at baseline, it was 17% in the previous study in Peru in 2002 [12], and did not differentiate the <−3.00 SD and −2.01 to −3.00 SD categories. Thus, we add to the literature that extreme short stature, such as less than −3 SD, can be a risk factor of future permanent dental caries in developed country like Japan, where a lack of nutrition or oral health literacy may not be a problem [19,20]. Further, the previous study used a limited sample size (N = 83), while the current study used a sufficient sample size (N = 3576); thus, it was possible to detect the association between extreme short stature at grade 1 and permanent dental caries at grade 6. Further, because the current study was population-based, we did not find an association between −2.01 SD and −3.00 SD and short stature and dental caries in the permanent teeth due to the low prevalence of permanent dental caries. The previous study in Peru showed a number of high DMFT, 5.10, while in current study, it was 0.42, although the point estimate showed risk (MR = 1.17).

There are some mechanisms that can explain the study findings. First, short stature is a marker of undernutrition in early childhood, which can be associated with permanent dental caries in pre-adolescence. Studies have reported the association between undernutrition and enamel hypoplasia in primary teeth [21,22,23], and enamel hypoplasia is prone to dental caries [8]. Second, protein–energy malnutrition can lead to salivary gland hypofunction in the primary dentition period, resulting in a decreased saliva volume, buffering capacity, and protein content [24,25,26]. The protective impact of saliva on the dental cavity is limited by inadequacies in vitamin A and D, as well as a shortage of protein and various micronutrients like vitamins, zinc, and iron [27]. Salivary gland hypofunction may lead to a high count of lactobacilli and Streptococcus mutans [28,29]. Moreover, longitudinal and cross-sectional studies have reported associations between malnutrition and enamel hypoplasia of the permanent teeth [30,31,32,33], as well as decreased salivary function in permanent dentition [34,35,36]. Early childhood infection and micronutrient deficiencies caused by malnutrition are possible causal mechanisms for developmental enamel defects in the permanent dentition [8]. Meanwhile, short stature is determined by multiple factors, including genetic factors, syndromes, or diseases during pregnancy or early childhood [37]. The cause of short stature may be the same as the cause of dental caries, and short stature could be a marker of childhood dental caries. Therefore, the association may not be causal. However, short stature at grade 1 can be used as a marker of high-risk groups for future permanent dental caries. Further research is needed to implement intervention studies to prevent permanent dental caries among those with a short stature of less than −3 SD at grade 1.

We found that children with short stature at grade 1, at 6–7 years old, had a higher risk of dental caries in their permanent teeth at grade 6 (11–12 years old). This finding suggests that the impact of childhood malnutrition on dental caries exists even in high-income countries. In Japan, children in low-income, dual-income, or single-parent families tend to eat prepared meals, such as fast food; eat out more often; or skip meals, [14,38] resulting in low-vegetable, low-nutrient, and unbalanced diets [39,40,41,42]. This differs from low- and middle-income countries, where malnutrition due to low socioeconomic status, early marriage, poor maternal nutrition, postnatal diet, and disease are determinants of malnutrition in children [43]. Policies that improve the nutritional status of children may reduce future dental caries and promote oral and general health.

This study has several limitations. First, the dental caries assessed by school dentists and height measurements by teachers were not calibrated for research purposes. Although the height measurement method is simple, it may lower inter-examiner reliability. Second, as for dental caries assessment, dental caries in children may have been underreported because an X-ray examination was not conducted during the school health checkup. Third, we did not have information on the fluoride utilization of the children. Water fluoridation is not implemented in Japan except for in US military bases, while fluoride toothpaste is widely available, with a market share of 91 percent in 2015 [44]. Therefore, the difference in fluorine exposure among the participants in this study would be small. Forth, genetic confounding between short stature and dental caries may exist, considering that GHR and BMP2 are associated with enamel dysplasia in the permanent teeth [45,46]. Finally, the present analyses used data from children who responded to both the baseline and follow-up surveys, which may result in selection bias. However, the characteristics were similar between the baseline and imputed samples (Supplementary Table S1).

## 5. Conclusions

This study found that short stature at grade 1 was associated with dental caries at grade 6 children in Japan. This association was robust after adjusting for potential confounders such as the children’s birth weight, socioeconomic status, or oral health behaviors. Further studies are required to elucidate these underlying mechanisms.

## Figures and Tables

**Table 1:** Height by demographic characteristics of participants at grade 1 (complete case; N = 2919).

		Height-for-Age Z-Score at Grade 1			
	Total N = 2919	≥−2.00 SD N = 2539; 87.0%	−2.01 SD–−3.00 SD N = 358; 12.3%	<−3.00 SD N = 22; 0.75%	
	N (%) or Mean (SD)	N (%) or Mean (SD)	N (%) or Mean (SD)	N (%) or Mean (SD)	*p*-Value
Age (months)	85.68 (3.31)	85.72 (3.29)	85.40 (3.45)	85.05 (3.43)	0.14
Sex					
Boy	1493 (51.2%)	1282 (50.5%)	197 (55.0%)	14 (63.6%)	0.14
Girl	1426 (48.9%)	1257 (49.5%)	161 (45.0%)	8 (36.4%)	
Number of erupted teeth at grade 6	23.76 (3.50)	24.0 (3.37)	22.3 (3.94)	21.5 (4.38)	<0.001
DMFT at grade 1	0.045 (0.30)	0.045 (0.30)	0.034 (0.21)	0.18 (0.59)	0.07
DMFT at grade 6	0.42 (1.10)	0.42 (1.09)	0.41 (1.12)	0.82 (2.02)	0.24
Birth Weight					
<2500 g	272 (9.3%)	190 (7.5%)	76 (21.2%)	6 (27.3%)	<0.001
2500–3999 g	2617 (89.7%)	2320 (91.4%)	281 (78.5%)	16 (72.7%)	
≥4000 g	30 (1.0%)	29 (1.1%)	1 (0.28%)	0 (0.00%)	
Annual household income at grade 1					
JPY <3.0 million	294 (10.1%)	254 (10.0%)	38 (10.6%)	2 (9.09%)	0.40
JPY 3.0–5.9 million	1205 (41.3%)	1032 (40.7%)	160 (44.7%)	13 (59.1%)	
JPY 6.0–9.9 million	908 (31.1%)	801 (31.6%)	102 (28.5%)	5 (22.7%)	
JPY 10.0+ million	250 (8.6%)	226 (8.90%)	24 (6.70%)	0 (0.00%)	
Unknown	262 (9.0%)	226 (8.90%)	34 (9.50%)	2 (9.09%)	
Mother’s educational attainment					
Less than high school	1061 (36.4%)	915 (36.0%)	140 (39.1%)	6 (27.3%)	0.46
Junior college or technical school	1248 (42.8%)	1079 (42.5%)	158 (44.1%)	11 (50.0%)	
University or more	589 (20.2%)	527 (20.8%)	57 (15.9%)	5 (22.7%)	
Others/Unknown	21 (0.7%)	18 (0.7%)	3 (0.84%)	0 (0.00%)	
Frequency of drinking SSB at grade 1					
<daily	2336 (80.0%)	2037 (80.2%)	281 (78.5%)	18 (81.8%)	0.73
≥daily	583 (20.0%)	502 (19.8%)	77 (21.5%)	4 (18.2%)	
Snack-eating habits at grade 1					
Controlled	2142 (73.4%)	1874 (73.8%)	251 (70.1%)	17 (77.3%)	0.31
Not controlled	777 (26.6%)	665 (26.2%)	107 (29.9%)	5 (22.7%)	

DMFT: decayed, missing, or filled permanent teeth; JPY: Japanese yen; SD: standard deviation; SSB: sugar-sweetened beverages.

**Table 2:** Results of Poisson regression analysis with robust standard error for DMFT at grade 6. (Multiple imputation applied; N = 3576).

	DMFT at Grade 6	Model 1		Model 2		Model 3	
	Mean (SE)	MR (95% CI)	*p*-Value	MR (95% CI)	*p*-Value	MR (95% CI)	*p*-Value
Height-for-age z-score at grade 1							
≥−2.00 SD	0.42 (0.02)	ref.		ref.		ref.	
−2.01 SD–−3.00 SD	0.42 (0.05)	1.18 (0.90, 1.55)	0.220	1.21 (0.92, 1.58)	0.172	1.17 (0.89, 1.54)	0.255
<−3.00 SD	0.81 (0.38)	2.07 (0.98, 4.36)	0.057	2.15 (1.00, 4.62)	0.050	2.18 (1.03, 4.64)	0.042
Age in months at grade 1	0.42 (0.02) ^a^	0.98 (0.96, 1.01)	0.124	0.98 (0.96, 1.01)	0.133	0.98 (0.96, 1.00)	0.118
Sex							
Boy	0.36 (0.02)	ref.		ref.		ref.	
Girl	0.48 (0.03)	1.22 (1.03, 1.45)	0.021	1.23 (1.03, 1.46)	0.019	1.24 (1.04, 1.47)	0.014
Number of erupted teeth at grade 6	0.42 (0.02) ^a^	1.09 (1.06, 1.12)	<0.001	1.09 (1.06, 1.12)	<0.001	1.08 (1.06, 1.11)	<0.001
DMFT at grade 1	0.42 (0.02) ^a^	1.91 (1.66, 2.20)	<0.001	1.92 (1.68, 2.20)	<0.001	1.95 (1.70, 2.25)	<0.001
Birth Weight							
<2500 g	0.40 (0.06)			0.86 (0.63, 1.18)	0.352	0.87 (0.64, 1.19)	0.390
2500–3999 g	0.42 (0.02)			ref.		ref.	
≥4000 g	0.53 (0.16)			1.28 (0.70, 2.32)	0.424	1.29 (0.72, 2.34)	0.394
Annual household income at grade 1							
JPY <3.0 million	0.57 (0.07)					ref.	
JPY 3.0–5.9 million	0.42 (0.03)					0.76 (0.58, 0.99)	0.042
JPY 6.0–9.9 million	0.39 (0.03)					0.76 (0.57, 1.02)	0.067
JPY 10.0+ million	0.35 (0.06)					0.71 (0.48, 1.05)	0.083
Unknown	0.43 (0.05)					0.71 (0.51, 1.01)	0.054
Mother’s educational attainment							
Less than high school	0.47 (0.03)					ref.	
Junior college or technical school	0.42 (0.03)					0.91 (0.74, 1.11)	0.352
University or more	0.34 (0.04)					0.82 (0.62, 1.07)	0.140
Others/Unknown	0.36 (0.19)					0.68 (0.24, 1.94)	0.473
Frequency of drinking SSB at grade 1							
<daily	0.40 (0.02)					ref.	
≥daily	0.50 (0.05)					1.11 (0.90, 1.37)	0.342
Snack-eating habits at grade 1							
Controlled	0.38 (0.02)					ref.	
Not controlled	0.52 (0.04)					1.29 (1.07, 1.57)	0.008

CI: confidence interval; DMFT: decayed, missing, or filled permanent teeth; JPY: Japanese yen; MR: mean ratio; SE: standard error; SSB: sugar-sweetened beverages. ^a^ mean number of dental caries in the entire sample.

## Data Availability

Data are not publicly available due to ethical reasons. Further enquiries can be directed to the corresponding author.

## Supplementary Materials

The following supporting information can be downloaded at https://www.mdpi.com/article/10.3390/ijerph21010105/s1: Table S1: Comparison of characteristics between the respondents to the baseline, followed, complete case, and imputed sample. Table S2. Results of Robust Poisson regression analysis for DMFT at grade 6. (complete case, N = 2919)
