# Supplementary material for: Association between Short Stature at Grade 1 and Permanent Teeth Caries at Grade 6 in Elementary School Children in Japan: A Population-Based Cohort Study
Source: Int J Environ Res Public Health. 2024 Jan 17;21(1):105. doi: 10.3390/ijerph21010105 (PMC10815877; doi:10.3390/ijerph21010105)
Supplement: Supplementary file 1 [file ijerph-21-00105-s001.zip › ijerph-2815867-supplementary.pdf]

Supplementary Table 1. Comparison of characteristics between the respondents to the baseline, followed, complete case, and imputed sample.

	Baseline sample	Followed sample	Complete case sample	Imputed sample
	n = 4291	n = 3576	n = 2919	n = 3576
	N (%) or mean (SD)	N (%) or mean (SD)	N (%) or mean (SD)	% or mean
**DMFT at grade 6**	NA	0.420 (1.09)	0.422 (1.10)	0.420
**Height-for-age z-score at grade 1**				
≥ -2.00 SD	3573 (87.3%)	2995 (83.8%)	2539 (87.0%)	87.4%
-2.01 SD - -3.00 SD	497 (11.6%)	407 (11.4%)	358 (12.3%)	11.9 %
< -3.00 SD	32 (0.8%)	25 (0.7%)	22 (0.8%)	0.7%
Missing	189 (4.4%)	149 (4.2%)	0 (0.0%)	0 (0.0%)
**Age in months at grade 1**	85.23 (3.57)	85.24 (3.58)	85.68 (3.31)	85.23
**Sex**				
Boy	2197 (51.2%)	1795 (50.2%)	1493 (51.2%)	50.3%
Girl	2088 (48.7%)	1777 (49.7%)	1426 (48.9%)	49.8%
Missing	6 (0.14%)	4 (0.1%)	0 (0.0%)	0 (0.0%)
**Number of erupted teeth at grade 6**	23.74 (3.53)	23.74 (3.53)	23.76 (3.50)	23.74
**DMFT at grade 1**	0.041 (0.27)	0.041 (0.28)	0.045 (0.30)	0.041
**Birth Weight**				
< 2500g	379 (8.8%)	320 (9.0%)	272 (9.3%)	9.2%
2500g - 3999g	3718 (86.7%)	3113 (87.1%)	2617 (89.7%)	89.7%
≥ 4000g	44 (1.0%)	38 (1.1%)	30 (1.0%)	1.1%
Missing	150 (3.5%)	105 (2.9%)	0 (0.0%)	0 (0.0%)
**Annual household income at grade 1**				
<3.0 million JPY	489 (11.4 %)	368 (10.3%)	294 (10.1%)	10.3%
3.0 - 5.9 million JPY	1718 (40.0%)	1439 (40.2%)	1205 (41.3%)	40.2%
6.0 - 9.9 million JPY	1284 (29.9%)	1112 (31.1%)	908 (31.1%)	31.1%
10.0+ million JPY	360 (8.4%)	315 (8.8%)	250 (8.6%)	8.8%
Unknown	440 (10.3%)	342 (9.6%)	262 (9.0%)	9.6%
Missing	0 (0.0%)	0 (0.0%)	0 (0.0%)	0 (0.0%)
**Mother's educational attainment**				
Less than high school	1455 (33.9 %)	1178 (32.9%)	1061 (36.4%)	36.4%
Junior college or technical school	1567 (36.5%)	1354 (37.9%)	1248 (42.8%)	42.6%
University or more	778 (18.1%)	646 (18.1%)	589 (20.2%)	20.3%
Others/Unknown	29 (0.7%)	22 (0.6%)	21 (0.7%)	0.8%
Missing	462 (10.8%)	376 (10.5%)	0 (0.0%)	0 (0.0%)
**Frequency of drinking SSB at grade 1**				
< daily	3093 (72.1 %)	2599 (72.7%)	2336 (80.0%)	79.8%
≥ daily	815 (19.0%)	655 (18.3%)	583 (20.0%)	20.2%
Missing	383 (8.9%)	322 (9.0%)	0 (0.0%)	0 (0.0%)
**Snack-eating habits at grade1**				
Controlled	2819 (65.7%)	2367 (66.2%)	2142 (73.4%)	72.8%
Not controlled	1084 (25.3%)	881 (24.6%)	777 (26.6%)	27.2%
Missing	388 (9.0%)	328 (9.2%)	0 (0.0%)	0 (0.0%)

Abbreviation: DMFT, decayed missed or filled permanent teeth; JPY, Japanese yen; SD, standard deviation; SSB, sugar-sweetened beverages;

Supplementally Table 2. Results of Robust Poisson regression analysis for DMFT at grade 6. (complete case, N = 2919)

	DMFT at grade 6	Model 1		Model 2		Model 3	
	Mean (SD)	MR (95% CI)	P-value	MR (95% CI)	P-value	MR (95% CI)	P-value
**Height-for-age z-score at grade 1**							
**≥ -2.00 SD**	0.42 (1.09)	ref.		ref.		ref.	
**-2.01 SD - -3.00 SD**	0.41 (1.12)	1.18 (0.88, 1.57)	0.276	1.19 (0.89, 1.60)	0.241	1.16 (0.86, 1.57)	0.318
**< -3.00 SD**	0.82 (2.02)	2.01 (0.89, 4.57)	0.095	2.06 (0.90, 4.74)	0.088	2.09 (0.93, 4.70)	0.076
**Age in months at grade 1**	0.42 (1.10) [a]	0.98 (0.96, 1.01)	0.270	0.98 (0.96, 1.01)	0.278	0.98 (0.96, 1.01)	0.258
**Sex**							
**Boy**	0.37 (1.04)	ref.		ref.		ref.	
**Girl**	0.48 (1.15)	1.17 (0.97, 1.41)	0.098	1.17 (0.97, 1.41)	0.099	1.18 (0.98, 1.42)	0.073
**Number of erupted teeth at grade 6**	0.42 (1.10) [a]	1.09 (1.06, 1.13)	<0.001	1.09 (1.06, 1.13)	<0.001	1.09 (1.06, 1.12)	<0.001
**DMFT at grade 1**	0.42 (1.10) [a]	1.88 (1.63, 2.18)	<0.001	1.89 (1.64, 2.18)	<0.001	1.91 (1.66, 2.21)	<0.001
**Birth Weight**							
**< 2500g**	0.42 (1.13)			0.90 (0.65, 1.27)	0.556	0.91 (0.65, 1.27)	0.577
**2500g - 3999g**	0.42 (1.10)			ref.		ref.	
**≥ 4000g**	0.40 (0.72)			0.98 (0.51, 1.87)	0.954	1.03 (0.54, 1.95)	0.926
**Annual household income at grade 1**							
**< 3.0 million JPY**	0.54 (1.35)					ref.	
**3.0 - 5.9 million JPY**	0.42 (1.01)					0.81 (0.59, 1.10)	0.172
**6.0 - 9.9 million JPY**	0.42 (1.19)					0.83 (0.60, 1.17)	0.293
**10.0+ million JPY**	0.32 (0.97)					0.67 (0.42, 1.06)	0.086
**Unknown**	0.43 (0.96)					0.75 (0.50, 1.11)	0.152
**Mother's educational attainment**							
**Less than high school**	0.47 (1.13)					ref.	
**Junior college or technical school**	0.42 (1.08)					0.88 (0.72, 1.08)	0.232
**University or more**	0.35 (1.08)					0.82 (0.62, 1.06)	0.150
**Others/Unknown**	0.38 (0.92)					0.75 (0.27, 2.06)	0.571
**Frequency of drinking SSB at grade 1**							
**< daily**	0.40 (1.07)					ref.	
**≥ daily**	0.51 (1.21)					1.12 (0.90, 1.41)	0.303
**Snack-eating habits at grade 1**							
**Controlled**	0.39 (1.06)					ref.	
**Not controlled**	0.51 (1.19)					1.26 (1.03, 1.55)	0.024

CI: confidence interval; DMFT: decayed, missed or filled permanent teeth; JPY: Japanese yen; MR: mean ratio; SD: standard deviation; SSB: sugar-sweetened beverages

[a] mean number of dental caries in the entire sample
